# Evaluation of The Expression Levels of Three Long Non-Coding RNAs in Multiple Sclerosis

**DOI:** 10.22074/cellj.2020.6555

**Published:** 2019-10-14

**Authors:** Afshin Moradi, Mahdis Rahimi Naiini, Nasrin Yazdanpanahi, Hossein Tabatabaeian, Fariba Nabatchian, Masoud Baghi, Mansoureh Azadeh, Kamran Ghaedi

**Affiliations:** 1. Department of Medical Laboratory Sciences, Faculty of Allied Medicine, Tehran University of Medical Sciences, Tehran, Iran; 2. Department of Biochemistry, Medical School and Kerman Physiology Research Center, Kerman University of Medical Science, Kerman, Iran; 3. Zist-fanavari Novin, Biotechnology Institute, Isfahan, Iran; 4. Department of Genetics, Falavarjan Branch, Islamic Azad University, Isfahan, Iran; 5. Division of Genetics, Department of Biology, Faculty of Sciences, University of Isfahan, Isfahan, Iran; 6. Department of Biochemistry, Yong Loo Lin School of Medicine, National University of Singapore, Singapore; 7. Department of Biology, School of Sciences, University of Isfahan, Isfahan, Iran; 8. Department of Cellular Biotechnology, Cell Science Research Center, Royan Institute for Biotechnology, ACECR, Isfahan, Iran

**Keywords:** Autoimmune Disease, Gene Expression Profiling, Long Non-coding RNA, Multiple Sclerosis, Neurodegenerative Disease

## Abstract

**Objective:**

Multiple sclerosis (MS) is a chronic disorder involving both inflammatory and neurodegenerative responses. Long non-coding RNAs (lncRNAs) have been had an emerging role as the biomarkers of different disorders, including autoimmune diseases. Previous studies have shown that NR_003531.3 (MEG3a), AC000061.1_201, and AC007182.6 play a role in the pathogenesis of human autoimmune diseases. However, the potential significance of these lncRNAs, as the diagnostic biomarkers of MS, has not been studied yet. We aimed to quantitatively evaluate the expression levels of NR_003531.3, AC000061.1_201, and AC007182.6 in peripheral blood samples of MS patients in comparison with healthy controls.

**Materials and Methods:**

In this case-control study, the blood samples from 20 MS patients and 10 healthy controls were collected. Total RNA was extracted, and the expression levels of three selected lncRNAs were quantitatively measured using the quantitative real time-polymerase chain reaction (qRT-PCR) method.

**Results:**

We detected a significant down-regulation in the expression of NR_003531.3 in MS patients, while no marked changes were observed in the expression of AC000061.1_201 and AC007182.6 in patients compared with controls. Based on the receiver operating characteristic (ROC) curve analysis, NR_003531.3 could discriminate MS patients from healthy subjects effectively. Regarding the prognosis of MS patients, NR_003531.3 is significantly and inversely correlated with the expanded disability status scale (EDSS).

**Conclusion:**

The potential role of NR_003531.3 lncRNA as a diagnostic biomarker to distinguish MS patients is proposed. Prognostically, NR_003531.3 correlates with lower disability rates in MS patients.

## Introduction

Multiple sclerosis (MS) is a chronic disorder involving both inflammatory and neurodegenerative responses. The origins of MS have been summarized as the environmental, genetic, and hormonal factors. Dramatic changes in human lifestyle have triggered a pervasive vitamin D deficiency worldwide. This phenomenon has elevated MS incidence (1). Along with vitamin D deficiency, estrogen hormone in females has synergistically worsened the immune tolerance and increased the number of MS cases (2).

Although the precise etiology of MS is still unrevealed, multiple genetic pathways have been identified to participate in the pathogenesis of MS, including HLA- DQB1*0602, HLA-DQA1*0102, HLA-DRB1*1501, and HLA-DRB5*0101 (3), as well as some microRNAs namely, miR-326 and miR-26a (4). MS disease is an inflammatory response with infiltrated activated monocytes, B and T cells into the central nervous system (5). Among them, T helper-17 (Th-17) plays a pivotal role in MS pathogenesis. Th-17 is a CD4^+^ T helper cell originated from Naïve CD4^+^ cells upon the expression and activation of interleukin-23 (IL-23), IL-6 and transforming growth factor-beta (TGF-β) (6- 8). Increased level of Th-17 cells has been reported in various human autoimmune diseases including MS (9), systemic lupus erythematosus (10), psoriasis (11), and rheumatoid arthritis (12). Th-17 cells impose their degenerative impact via up-regulating different cytokines such as granulocyte/macrophage colonystimulating factor (GM-CSF), IL-21, IL-17, and IL 22, which in turn, leads to the tissue injuries.

Several studies have identified numerous potential biomarkers that can predict the MS disease, as well as its progression. Among the applicable biomarkers, long non-coding RNAs (lncRNAs) contribute to the pathogenesis of many disorders such as MS (13). lncRNAs are >200-nucleotide non-coding transcripts expressed ubiquitously from the genome. AC007182.6 has been recently shown to be involved in Th-17 differentiation. This lncRNA is co-expressed with its nearby gene, *BATF*, which has a significant role in determining the naïve cell fate to be differentiated into Th-17 cells (14).

Furthermore, the overexpression of Homo sapiens maternally expressed 3a (MEG3a), also known as NR_003531.3, has been reported in the development of CD4+ T cells in autoimmune diseases. This excessive expression is strongly linked to the lowered percentage of Th-17 cells (15). The fundamental role of another lncRNA, named AC000061.1-201, has been revealed in rheumatoid arthritis. This lncRNA is significantly overexpressed in the peripheral blood mononuclear cells of these patients and tightly associated with the serum level of IL-6 and tumor necrosis factor-alpha (TNF-α) (16). Albeit these lncRNAs have substantial roles in the pathogenesis of autoimmune disorders, mainly through regulating the population of Th-17 cells, no study has been conducted to evaluate their significance in MS disease. Therefore, we aimed to quantitatively study the expression levels of NR_003531.3, AC000061.1-201, and AC007182.6 in the peripheral blood samples of the patients with MS disease. This study may help nominate the lncRNA(s), differentially expressed in MS patients, as prognostic molecules especially in MS subclinical cases.

## Materials and Methods

### Patients and samples

**Fig 1:**
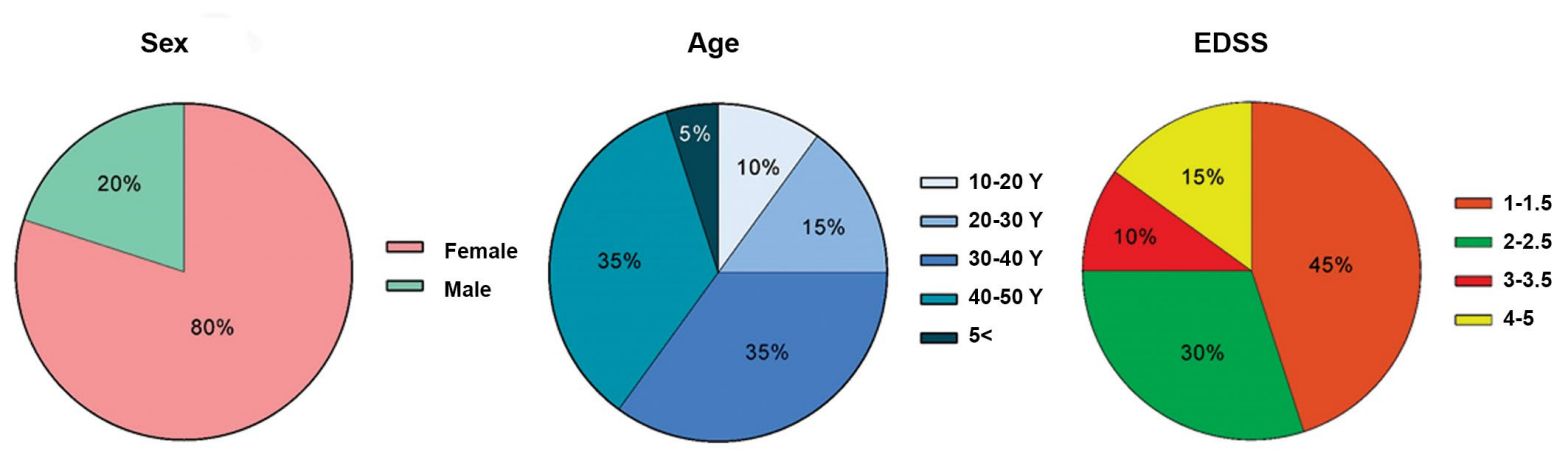
The characteristics of multiple sclerosis (MS) patients. The number of individuals as well as their gender, age, and the Expanded Disability Status Scale (EDSS) scores is illustrated.

The case-control study was carried out to compare lncRNA expression levels in 20 Iranian relapsingremitting MS (RRMS) patients who had not taken any kinds of MS drug. The diagnosis of RRMS has been made based on the revised McDonald’s criteria (17). The control group comprised of 10 healthy sexmatched volunteers whose neurological disorders were ruled out. Both groups of men and women, with the age range of 10 to 55, who signed the written informed consent, were included in the study. The participants with the history of autoimmune and/or neurological diseases, as well as diabetes mellitus, were excluded from the investigation. The characteristics of RRMS patients are summarized in Figure 1. In this research, 5 ml of peripheral blood was collected from all participants in EDTA-containing tubes.

All procedures performed in studies involving human participants were in accordance with the ethical standards of the Ethics Committee of Tehran University of Medical Sciences, Iran, and with the 1964 Helsinki declaration and its later amendments or comparable ethical standards. All participants signed the informed consents.

### RNA extraction

Total RNA was isolated with GeneAll Hybrid-RTM blood RNA extraction kit. The RNA concentration was estimated using Nanodrop Spectrophotometer (ND-1000, ThermoFisher, MA, USA). Purified RNA was stored at -80˚C for further steps.

### cDNA synthesis and quantitative real time-polymerase chain reaction

RNA was treated with DNase I prior to cDNA synthesis. The cDNA was performed using cDNA Synthesis Applied by Universal cDNA Synthesis Kit (ParsGenome, Iran) based on the manufacturer’s protocol. The synthesized cDNAs were stored at -20˚C until the polymerase chain reaction (PCR). The Oligo 7 software (Molecular Biology Insights, Inc., CO, USA) was employed for designing the specific primers, which are listed in Table 1. Quantitative real time- polymerase chain reacttion (qRT-PCR) reactions were performed on the ABI PRISM 7500 instrument (Applied Biosystems, USA). All reactions were carried out in triplicate. qRT-PCR data were assessed according to the 2^-ΔΔCT^ method (18). The corresponding lncRNA C_t_ values were normalized against *GAPDH*, as a reference gene.

**Table 1:** The sequence of the primers


lncRNA	Primer sequence (5´-3´)

NR_003531.3	F: TGGCATAGAGGAGGTGAT
	R: GGAGTGCTGTTGGAGAATA
AC000061.1_201	F: ATGCTGCTATGCTTCCC
	R: GCTTCTGTAGTTCGGTCTT
AC007182.6	F: TGTGTTACTCAGCGTCCTA
	R: CGTATTGAGAGCGTGTGTT


lncRNA; Long non-coding RNA.

###  Statistical analysis 

Statistical analyses were performed by the GraphPad Prism 7 (CA, USA). The Mann-Whitney U test was carried out to analyze the quantitative expression level of lncRNAs between patients and healthy groups. Spearman’s rank correlation test was performed to evaluate the possible correlation between the relative expression levels of lncRNAs and the clinical data. The P<0.05 were considered statistically significant.

## Results

### Expression levels of lncRNAs in RRMS patients and controls

**Fig 2:**
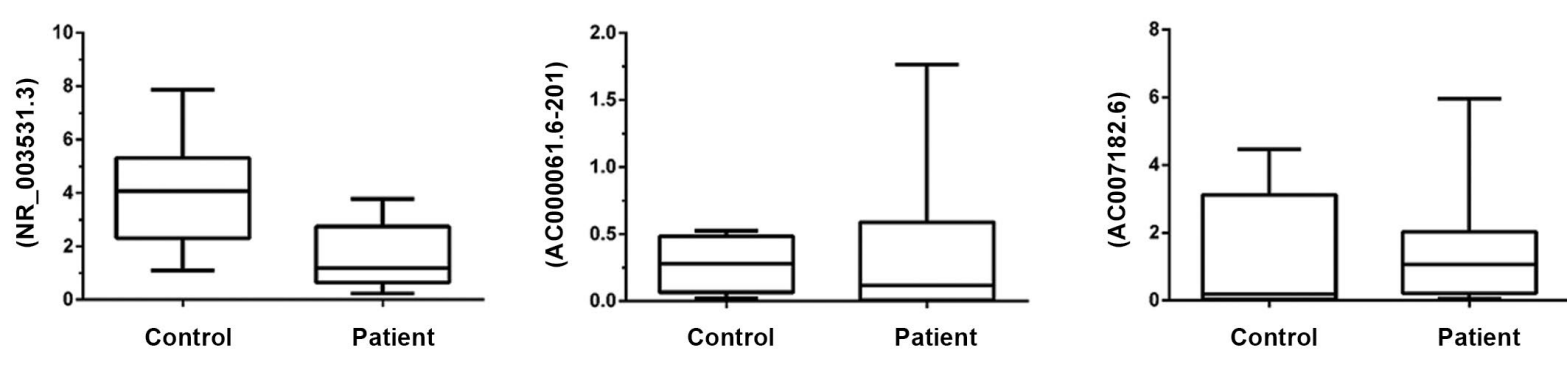
Expression level of NR_003531.3 (***; P=0.0003), AC000061.6-201 (P=0.99), and AC007182.6 (P=0.1619) in multiple sclerosis (MS) and healthy groups. While the previous long non-coding RNA (lncRNAs) had no significant differences, NR-003531.3 expression shows a significant increase in MS patients as compared to healthy controls.

The expression levels of three lncRNAs including NR_003531.3, AC000061.1-201, and AC007182.6 were quantitatively assessed using the qRT-PCR method in MS and healthy groups. Statistical analyses showed that there was a significant down-regulation in the expression of NR_003531.3 in RRMS patients, while no significant difference was observed concerning the expression levels of AC000061.1-201 and AC007182.6 in patients as compared to controls (Fig .2).

## NR_003531.3 discriminated MS patients from healthy controls

**Fig 3:**
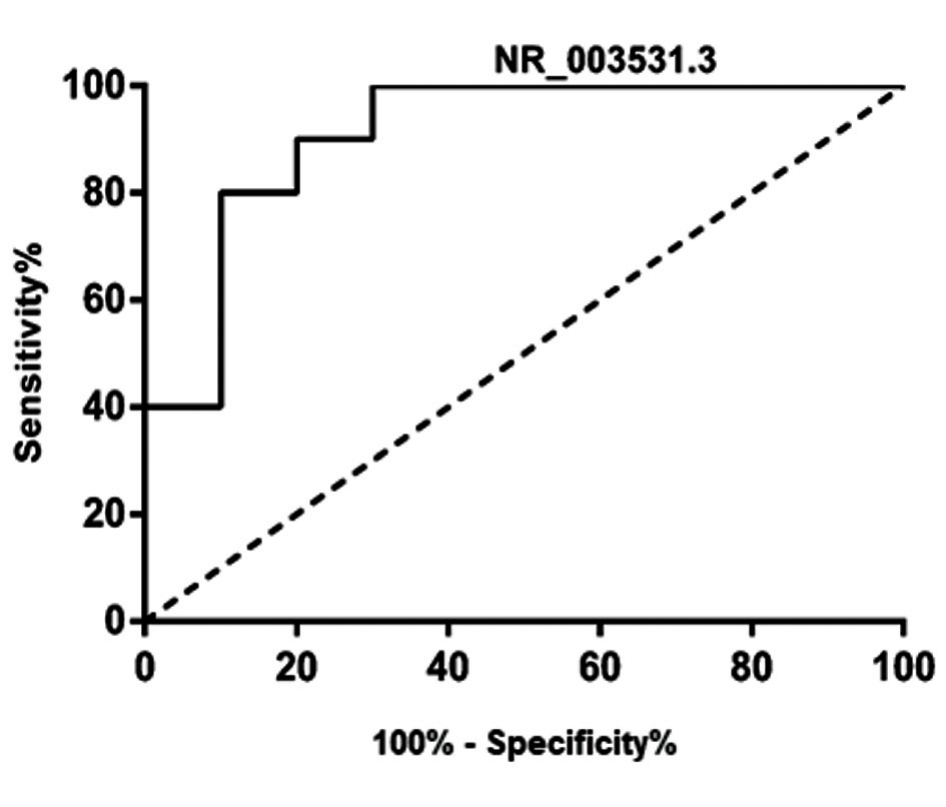
The receiver operating characteristic (ROC) curve of healthy/ patients analyzed for the expression level of NR-003531.3. NR-003531.3 is adequately potent to discriminate between healthy subjects and MS patients with the area under the curve (AUC) of 0.91 (P=0.0019).

To assess the potential of NR_003531.3, as a diagnostic biomarker for distinguishing the health/ disease status, the expression level of this lncRNA was analyzed by the receiver operating characteristic (ROC) analysis in both groups. The ROC curve analysis indicated that NR_003531.3 could effectively discriminate RRMS patients from healthy individuals with an area under the curve (AUC) of 0.90 (P=0.0019) (Fig .3). This finding may propose the NR_003531.3 as a potential screening tool.

## NR_003531.3 expression level is negatively correlated with the expanded disability status scale

**Fig 4:**
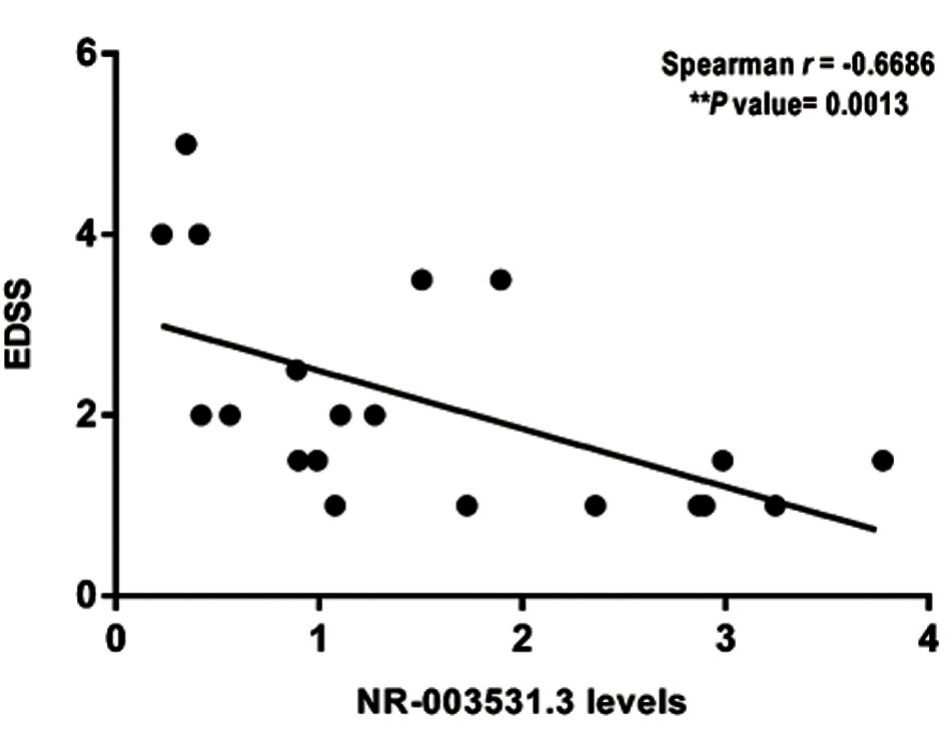
The correlation study. NR_003531.3 is negatively associated with the Expanded Disability Status Scale (EDSS) in multiple sclerosis (MS) patients.

In order to explore the prognostic value of NR_003531.3, the correlation between NR_003531.3 and the level of disability caused by MS disease was investigated. To this aim, the expression level of NR_003531.3 was evaluated based on the EDSS scores among 20 RRMS patients. It was demonstrated that there was a significant negative correlation between NR_003531.3 expression levels and the EDSS scores, r=-0.669 (P=0.0013). It implies that the higher expression of NR_003531.3 lncRNA was linked with the lowered disability rate in MS patients (Fig .4).

## Discussion

Non-coding RNAs have critical indications in a variety of human disorders including cancer (19-21), cardiovascular diseases (22), diabetes (23), and autoimmune diseases (24). LncRNAs are the emerging type of non-coding RNAs, playing crucial roles in the pathogenesis of human autoimmune diseases, especially MS (13). In this study, we screened the expression levels of three lncRNAs with implications in other human autoimmune diseases, in order to understand whether they are differentially expressed in MS versus healthy subjects.

NR_003531.3 is known to inhibit the miR-125a-5p expression in CD4+ T cells which, in turn, results in the lower percentage of Th-17 cells in immune thrombocytopenic purpura (15). More importantly, NR_003531.3 has been reported to be critical as a functional lncRNA, which plays a significant role as a competing endogenous RNA (ceRNA). NR_003531.3, indeed, competes with programmed cell death 4 (PDCD4) mRNA thereby binding to miR-21. The ultimate consequence of this phenomenon is ischemic neuronal death (25). It has been shown that NR_003531.3 is normally expressed in the nucleus accumbens of normal human brain tissue, while it shows a drastic up-regulation in the brain of heroin abusers (26), which may cause a neurodegenerative condition. All these data support the notion that NR_003531.3 might be necessary for the regulation of the pathway, thought to be involved in the modulation of the immune system and central nervous system.

In this study and for the first time, we have demonstrated the expression level of three lncRNAs including NR_003531.3, AC000061.1-201, and AC007182.6 in MS patients. The evaluation of extensive alterations that occur in the expression of lncRNAs during the immune response could result in designing novel diagnostic and therapeutic approaches for MS patients. Based on the analysis, NR_003531.3, but not other lncRNAs, showed a significant down-regulation in RRMS patients. It strongly suggests that NR_003531.3 could decrease the risk of developing MS by reducing the percentage and activity of Th-17 cells.

Additionally, the ROC curve analysis showed that NR_003531.3 is effectively capable and potent to distinguish between RRMS and healthy individuals. These findings suggest that NR_003531.3 might be considered a novel potential biomarker for the diagnosis of MS patients. However, since MS patients are mainly diagnosed by the clinical and imaging findings, the applicability of NR_003531.3 would be useful when its altered expression is assessed in relapse/acute phase of the disease in comparison with the remission stage. This hypothesis can be examined by the determination of the expression level of NR_003531.3 in MS patients when they experience the relapse phase in comparison with those at the remission stage.

The correlation analyses between NR_003531.3 lncRNA expression levels and EDSS scores of MS patients revealed a significant negative correlation between NR_003531.3 levels and the EDSS, denoting that the lower expression of NR_003531.3 is associated with the higher disability rate in MS patients. This finding highlights the prognostic value of NR_003531.3 in MS disease.

As NR_003531.3 has a differential expression in MS patients, as compared to controls, this lncRNA might be participating in the pathogenesis of MS. This hypothesis could be expanded by *in vitro* and *in vivo* studies using NR_003531.3 expression plasmid and/or specific siRNAs. As modulating the percentage of Th-17 cells might be the main function of NR_003531.3, incorporating the quantitative studies to analyze the population of Th-17 cells, downstream of NR_003531.3, could gain an insight on the mechanistic role of this lncRNA in MS disease.

A number of altered lncRNAs in MS have been reported. Based on the microarray analysis in MS patients, Zhang et al. (27) have shown that ENSG00000231898.3, XLOC_009626, and XLOC_010881 underwent up-regulation, while ENSG00000233392.1, ENSG00000259906.1, and lncRNA XLOC_010931 exhibited a lower amount in MS patients. Eftekharian et al. (13) revealed that PVT1 and FAS-AS1 lncRNAs had lower expression as compared with healthy individuals, whereas THRIL had significantly higher expression in RRMS patients. Moreover, a lncRNA PCR array-based study showed the down-regulation of NRON and TUG1 in MS patients (28). Unlike the latter study, TUG1 along with NEAT1 and PANDA has been reported to be overexpressed in MS patients (29), reflecting the significance of validating the results at least for TUG1 lncRNA. Besides, Pahlevan Kakhki et al. (30) indicated that HOTAIR lncRNA underwent up-regulation in MS patients. In contrast to these studies, we have evaluated the diagnostic and prognostic values of lncRNAs that were earlier mentioned.

In brief, we have demonstrated the down-regulation of NR_003531.3 in MS patients. Based on the ROC curve analysis, this lncRNA is capable of discriminating the MS patients effectively. Prognostically, the lower expression of NR_003531.3 is linked with a poorer prognosis in MS disease.

This study lacked sufficient sample size. Therefore, to confirm the results, further studies are required to examine the expression level of NR_003531.3 in a larger population of MS and control groups, as well as a separate group of MS patients to test whether the disease stage (relapse/ remission) influence the expression of NR_003531.3. Moreover, animal models of MS could be employed to corroborate the results of our study. As shown in our study that NR_003531.3 is downregulated in MS patients, the overexpressing this lncRNA would affect the onset or the prognosis of MS in murine models.

## Conclusion

NR_003531.3 is significantly downregulated in the peripheral blood of MS patients. This lncRNA can discriminate MS patients from healthy controls. These findings propose the potential diagnostic value of NR_003531.3 lncRNA. Moreover, NR_003531.3 is inversely correlated with disability scores of MS patients, suggesting the potential prognostic role of this lncRNA on MS disease course.
